# The Perkins-Hyatt or Celluloid Base

**Published:** 1872-02

**Authors:** W. H. Eames


### ARTICLE VIII.

### *The Perkins-Hyatt or Celluloid Base.*

BY W. H. EAMES, D. D. S.

In the April number of the *Journal*, of the current year, we called the attention of our readers to this new base, and published, as an item of interest, the circular issued by the patentees, adding a word of caution against a too hasty adoption of what was then an untried experiment. Sufficient time has now elapsed to prove, we think, the utter worthlessness of this material as a base for artificial teeth, and agreeable to a promise made when we took up the editorial quill, we publish the following list of experimental cases with this base, with the results, as our own experience, and upon which, in part, we base our opinions of its merits.

Case No. 1. Mrs. P., full upper set gum teeth; returned in three weeks, so badly shrunk that the heels of the plate would not pass over the plaster model by one-eighth of an inch. The bicuspid blocks, on both sides, drawn inwards, so that the upper edge of the gum was entirely out of the socket formed by the rim of the plate above the blocks. Color of plate, pink. Put up in boiling oil.

Case No. 2. Mrs. H., full upper plate plane teeth; returned in two weeks, shrunk so that the plate would not touch centre of the palate by one-fourth of an inch. Softened the plate and readapted it to the plaster model. Returned in one week, worse than ever. Color of plate, dark brown; boiled in oil. Plate inserted soon after extracting the natural teeth.

No. 3. Mrs. H., full upper temporary set; returned in two weeks, so much out of shape, that it could not possibly be worn. Color of plate pink; boiled in oil.

No. 4. Mrs. R., full upper and lower set; returned in four weeks, shrunk so that it was impossible to wear them. The heels of the under plate one-fourth of an inch nearer each other than when first made.

No. 5. Mrs. E., full set of upper and lower gum teeth; returned in three months, all drawn out of shape; the under teeth drawn inwards, throwing the edges of the gum out of the rim, and the plates so curled up that the posterior molars would not articulate by at least one-fourth of an inch. The worst case of shrinkage and twisting in the lot.

No. 6. Mrs. R., full set upper and lower. Put up in June, is still worn, but a very poor fit. Color, light pink; boiled in oil.

No. 7. Miss L., full upper and lower. Put up in June; still worn; plate without any coloring material. Translucent.

No. 8. Miss I., full set upper; returned in three weeks. Made a new plate of the improved material; returned in six weeks, entirely out of shape.

No. 9 Miss K., upper set; put up in May; returned in six weeks. Made a new plate; second plate returned in eight weeks, worthless.

No. 10. Mrs. E., full upper and lower gum teeth; made in June; returned in three months; blocks out of place and the plate all out of shape.

No. 11. Miss C., partial plate; returned in three weeks, worthless.

No. 12. Miss I., partial; returned in four weeks, worthless.

No. 13. Mr. H., full set; made in June; returned in four weeks; second plate of the improved material returned in six weeks, worthless.

No. 14. Mrs. B., full set temporary; returned in four weeks, worthless.

No. 15. Mrs. B., full lower set temporary ; made in May. Still worn, but badly out of shape.

Ten other cases, returned as worthless.

We think comment on the above record unnecessary ; it speaks for itself. Of all the cheap materials which have been palmed off upon the profession, as bases for artificial teeth, this is undoubtedly the poorest.

We have tried the different qualities of this material, and find but little difference in the result. The plates having the least camphor combined with them being undoubtedly the best; but they all contain too mueh of this element. Unless the manufacturers can overcome the shrinkage and warping of this material, it can be of but litile use.

In the last number of the *Dental Cosmos* may be seen a full page advertisement, recommending very highly the celluloid base, and stating emphatically that all objections urged against it have been overcome. We have received plates, since this advertisement appeared, that do not differ materially from those manufactured three or four months since.

'Tis said, there are tricks in all trades, and this trick of puffing, by means of circulars, purporting to be issued by disinterested parties—men well known to the profession—and solely for the benefit of brother dentists, who are supposed to be anxious inquirers after truth, and by laudatory puffs and flaming advertisements, in our dental journals, is one with which our profession is largely cursed at the present day, and one which has cost many a poor dentist, living remote from our large cities, dollars for which he has received no benefit. "We know how it is ourself."—*Missouri Dental Journal.*

